# Observational Study of the Clinical Impact of E-scooter Injuries at a Major Trauma Centre

**DOI:** 10.7759/cureus.68788

**Published:** 2024-09-06

**Authors:** Arsany Metry, Nauman Manzoor, Kanishka Wattage, Fahad Hussain, Kerollos Khilla, Arnab Sain, Zain Sohail

**Affiliations:** 1 Trauma and Orthopaedics, Worthing Hospital, University Hospitals Sussex NHS Foundation Trust, Worthing, GBR; 2 Trauma and Orthopaedics, St Mary's Hospital, Isle of Wight NHS Trust, Isle of Wight, GBR

**Keywords:** electric scooter accidents, e-scooter injuries, major trauma, open injury, public transport

## Abstract

Background

Due to the increased use of e-scooters in the UK, associated injuries have increased, and its cost has increased as well. Worldwide data on injuries related to e-scooter use are relatively limited, owing to the short duration since their first introduction to the public.

Aim

There has been an increase in using e-scooters as a method of transport recently. It was noticed that the severity and frequency of its injuries are increasing as well. This study focuses on the frequency of e-scooter-associated injuries, especially open fractures, and to evaluate its burden on the health system.

Methods

Data on e-scooter injuries were extracted from our trauma database from November 2017 to July 2022. Patients’ notes and images were reviewed. Outcome measures were the type of injuries: site of bony fractures, closed vs. open fracture (using Anderson and Gustilo classification), number of operations, complications, and length of hospital stay.

Results

The number of patients enrolled was 104. The mean age was 34.3 years, and 78.8% (n = 82) were male. The main mechanism of injury was riding (91%, n = 95) vs. hitting by an e-scooter (9%, n = 9). Injured patients were more likely not to wear helmets (82% of total injured patients). Patients with bony injuries were 65.4% (n = 68), with 22.1% (n = 15) of them being open fractures. The most common bony injuries were lower limb-only fractures at 45.6% (n = 31), then upper limb-only injuries at 39.7% (n = 27). Combined upper and lower limb cases were 8.8% (n = 6), and pelvic injuries were 5.9% (n = 4). Head and neck injuries composed 23.1% (n = 24) of the reported injuries, including intracranial haemorrhages (9.6%, n = 10), extensive traumatic brain injury (3.8%, n = 4), haematoma/lacerations (3.8%, n = 4), cervical spine fractures (1.9%, n = 2), and skull fractures (1.9%, n = 2). The mean duration of hospital stay was 8.6 days, and 9.6% (n = 10) of patients needed intensive therapy unit (ITU) admission. The number of patients presented as trauma calls was 55.8% (n = 58). Patients who needed surgical intervention either under orthopaedics or other specialties were 52.9% (n = 55) and 21.2% (n = 22) of total patients had complications either due to surgical intervention or a long hospital stay.

Conclusion

E-scooter riding can lead to serious injuries that can end with limb- or life-threatening conditions. The most common demographic characteristics were adults in their early 30s. There should be more emphasis on wearing protective gear like wrist and elbow guards in addition to helmets. Future prospective studies with larger cohorts across multiple regions and hospitals are necessary to truly characterize the nature and cost of e-scooter injuries.

## Introduction

Due to the increased use of e-scooters in the UK, associated injuries have increased, and their cost reached about £1000.00 per patient [1]. E-scooters have a lot of advantages that have started to be considered after COVID-19 like being easy to apply social distancing, reducing congestion, easy to use, and inexpensive compared to other public transport means.

Worldwide data on injuries related to e-scooter use are relatively limited, owing to the short duration since their first introduction to the public. In the US, the population-adjusted occurrence of e-scooter-associated accidents multiplied from 1.53 per 100,000 in 2014 to 9.22 per 100,000 in 2019 [2]. It was noticed that the severity and frequency of its injuries are increasing as well.

The aim of the study is to focus on the frequency of e-scooter-associated injuries, especially open fractures, and to evaluate its burden on the health system.

This article was previously presented as a poster abstract at the Association of Surgeons in Training (ASiT) 47th Annual Conference on May 4, 2023.

## Materials and methods

Data collection

Patients presenting to the emergency department of a major trauma centre in London, United Kingdom, with an e-scooter injury were included. Data were extracted from our trauma database using electronic hospital records which recorded scooter or electric scooter injuries.

All medical records were reviewed for eligibility by a trauma clinician, and data were collected by scanning patients’ medical histories, outpatient documentation, surgery reports, and imaging reports.

Inclusion criteria

Acute traumatic cases presented to accident and emergency (A&E) due to an e-scooter injury from November 2017 to July 2022 were included in the study.

Exclusion criteria

Patients who had injuries from non-e-scooter accidents (like push scooters) were excluded.

Ethical statement

This was a retrospective observational study and did not require ethical approval according to the NHS Health Research Authority or informed consent; hence, no patients have been contacted or asked to participate.

Outcome measures

All identified episodes were retrospectively reviewed, and a spreadsheet was completed by assessing patient demographics (e.g., age, sex). Use of helmet and mechanism of injury (riding vs. hitting by) were included. Specific injuries were divided by area of the body, and the type of injury was recorded, including classifying open fractures according to the Anderson and Gustilo classification.

A number of operations and complications were recorded. Admission characteristics included how the patient was managed when he/she came in (trauma call, reviewed in A&E) and hospital stay. Teams were involved, and if patients needed intensive therapy unit (ITU) admission, they were recorded. 

Statistical analysis

Descriptive analyses were performed. Absolute numbers and percentages for variables were reported. 

## Results

There were 104 patients enrolled, and their average age was 34.3 years. In total, 78.8% (n = 82) were male and 22 were female. A total of 58 patients (55.8%) were trauma calls. The majority of presentations were orthopaedic injuries, with 65.4% (n = 68) and 22.1% (n = 15) of them being open fractures. Head and neck injuries came next, accounting for 23.1% (n = 24) of the injuries that were reported.

**Figure 1:**
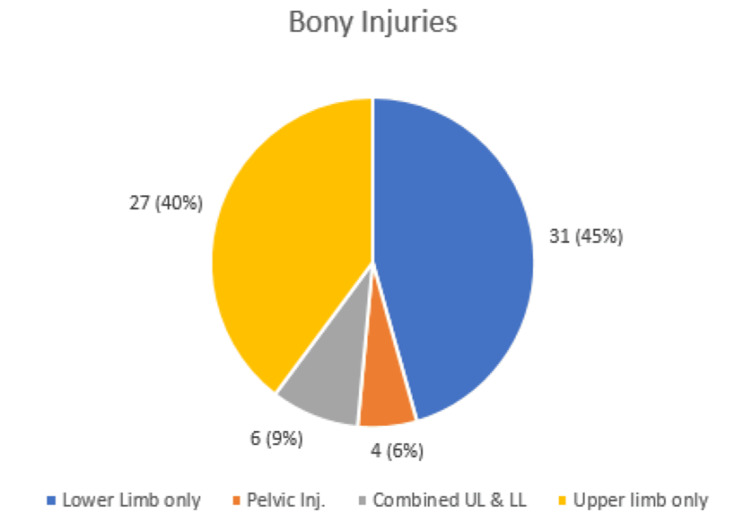
Distribution of orthopaedic injuries Inj: injury; UL: upper limb; LL: lower limb

The distribution of orthopaedic injuries is illustrated in Figure 1, with most of them (31 injuries, 45%) involving lower limbs only, followed by upper limb only injuries (27 injuries, 40%) and combined upper and lower limb injuries (six injuries, 9%). The least common presentation was pelvic injuries (four injuries, 6%).

Head and neck injuries comprised 23.1% (n = 24) of the reported injuries, and their distribution is shown in Table 1. Intracranial haemorrhages were the most common head and neck injuries (10 patients, 9.6%), followed by extensive brain injuries (four patients, 3.8%), extra-cranial haematomas and lacerations (four patients, 3.8%), and fractures (four patients, 3.8%).

**Table 1:** Head and neck injury distribution

Intracranial haemorrhages	9.6%, n=10
Extensive traumatic brain injury	3.8%, n=4
Haematomas/lacerations	3.8%, n=4
Cervical spine fractures	1.9%, n=2
Skull fractures	1.9%, n=2

Injured patients were more likely not to wear helmets (82% of total injured patients). Fifty-five patients (52.9%) required surgical intervention under either orthopaedic or other speciality. Ten patients had to be admitted to the ITU. The mean duration of hospital stay was 8.6 days. Twenty-two patients (21.2% of total patients) experienced complications as a result of lengthy hospital stays or surgical procedures. 

## Discussion

There are a number of benefits to using an e-scooter as a method of transport, especially in big cities. However, some riders can sustain high-energy injuries with significant medical and financial burdens. Going in line with the literature, this study shows an increasing frequency of serious injuries due to e-scooters. Thus, there should be more legislation for their usage, which might reduce unnecessary accidents.

In this study, it was found that 6.3% of riders injured were under the age of 18. This is a high percentage compared to the literature (5.6% of a systematic review [3]). Most injuries were isolated to the rider, in keeping with other literature, of which one of them showed that 53 patients out of 54 total patients were riders [4].

The lack of helmet use was consistent across all the previous studies, with most reporting <2% [3], while this study showed an increased rate of helmet use among riders (18%).

There is limited data in the literature about injuries that need surgical intervention. This study showed about 52% underwent surgeries. Like many other studies, this study identified a large proportion of extremity injuries, with lower limb injuries being more common. Open fractures with e-scooter injuries were not highlighted before in the literature. It was found to be significant in this study (22.1%), and all of them needed surgical intervention.

Interestingly, but not surprisingly, the most common injury reported was fracture, followed by injury involving the head and neck. This indicates how serious e-scooter injuries are.

In this study, a higher number of patients required surgical intervention; this is more than previously reported (27.6-29%) [1]. Our ITU admission rate is higher than the other UK study. [5]

Trivedi et al. stated that although the absolute number of admissions was relatively low, there are significant logistical and financial burdens to emergency departments [6]. One of the studies reported that the mean billing cost of e-scooter injuries was USD 95.710 [7]. This study showed as well that more than half of patients with e-scooter injuries needed surgical intervention (52.9%, n = 55).

Limitations

This study was based on retrospective data collection, leading to selection bias. Costs were not calculated, which gives a better idea of the financial burden of these types of injuries on the health system. Secondary healthcare outcomes, including time off from work or other benefits, were not considered.

## Conclusions

Riding e-scooters can result in severe injuries that may lead to life-threatening conditions, particularly open fractures. The typical demographic profile involved adults in their early thirties.

It is crucial to prioritise the use of protective gear such as wrist and elbow guards in addition to helmets. Conducting future studies with larger sample sizes across different regions and medical facilities is essential for accurately understanding the characteristics and economic impact of e-scooter injuries.
